# Treatment of Morbid Dentition

**Published:** 1873-11

**Authors:** E. D. Drake


### ARTICLE XI.

### *Treatment of Morbid Dentition.*

BY E. D. DRAKE, M. D.

I had occasion last January to prescribe for the patient of another physician, a boy æt. one year, suffering from derangement of the stomach and bowels while teething; his stomach was very irritable, bowels moving frequently, head hot and temper fretful; he had some fever, but temperature not remembered. He had been taking for several days a mixture of paragoric, lime water and milk, without any permanent benefit. Ordered.

| | | | |
|---|---|---|---|
| ℞. | Pot. bromidi pulv. | grs. xv. | |
| | Pot. nit. pulv. | grs. xij. | |
| | Zinci. oxidi. | grs. ij. | M. |

Divide in chart No. vi. S. One powder every four hours until bedtime.

Added nit. pot. on account of the fever; in twenty-four hours he was greatly improved; stomach and bowels quiet, fever abated and temper playful; continued brom. pot. and he had no further trouble. At the next eruption of teeth he had the same symptoms, which yielded readily to the same prescription without any special treatment directed to the alimentary canal, the vomiting and purging being restrained by quieting the nervous irritability, in other words removing the cause. The above treatment is based on the idea that the anorexia, vomiting and purging are reflex symptoms dependent on irritability of the nerve centres, which unchecked is liable to eventuate in convulsions, congestion or inflammation of the brain, or inanition from derangement of the digestive function.

In my opinion the treatment is safe, and can be kept up some time, and is really more valuable in the prevention than the cure of bad symptoms. I have not used this treatment extensively, but am pleased with it thus far, and emboldened to ask a trial of it in this disease in the way of prophylaxis and cure; the oxide of zinc is combined in the above from its supposed sedative effects, and I should think that a definite compound of bromine and zinc would be useful and convenient in various nervous affections. The "child of the period" is an interesting study, and needs attention as well as the girls and boys of the period; begotten of nervous parents he has a double inheritance of nervous predisposition, with a lack of physical development in other respects.—*Med. and Surg. Reporter.*

